# A systematic review of mixed studies on malaria in Colombia 1980–2022: what the “bifocal vision” discovers

**DOI:** 10.1186/s12889-023-16098-5

**Published:** 2023-06-17

**Authors:** Jaiberth Antonio Cardona-Arias, Walter Salas-Zapata, Jaime Carmona-Fonseca

**Affiliations:** 1 School of Microbiology, University of Antioquia, Medellín, Colombia; 2 Research group “Salud y Comunidad César Uribe Piedrahíta” School of Medicine, University of Antioquia, Medellín, Colombia

**Keywords:** Malaria, Paludism, Systematic review, Colombia

## Abstract

Mixed methods are essential in public health research and malaria control, because they allow grasping part of the complexity and diversity of the factors that determine health-disease. This study analyzes the mixed studies on malaria in Colombia, 1980–2022, through a systematic review in 15 databases and institutional repositories. The methodological quality was assessed with *Mixed Methods Appraisal Tool* (*MMAT*), *STrengthening the Reporting of OBservational studies in Epidemiology* (*STROBE*), and *Standards for Reporting Qualitative Research* (*SRQR*). The qualitative and quantitative findings were grouped into a four-level hierarchical matrix. The epidemiological profile of malaria morbidity, from traditional epidemiology, has been sustained by environmental problems, armed conflict, individual risk behaviors, and low adherence to recommendations from health institutions. However, the qualitative component reveals deeper causes that are less studied, of greater theoretical complexity, and that reflect challenges to design and implement health interventions, such as socioeconomic and political crises, poverty, and the neoliberal orientation in the malaria control policy; the latter reflected in the change in the role of the State, the fragmentation of control actions, the predominance of insurance over social assistance, the privatization of the provision of health services, the individualistic and economistic predominance of health, and low connection with popular tradition and community initiatives. The above confirms the importance of expanding mixed studies as a source of evidence to improve malaria research and control models in Colombia, and to identify the underlying causes of the epidemiological profile.

**Supplementary Information:**

The online version contains supplementary material available at 10.1186/s12889-023-16098-5.

## Introduction

Malaria is a public health problem that mainly affects poor populations [1]. There is a high number of malaria cases in America, mainly in Brazil, Nicaragua, and Venezuela [2]. There have been around 65 thousand cases per year in the last five years in Colombia, mostly in Afro-Colombians and indigenous people [3]. The transmission pattern is unstable with variable interannual and inter-monthly prevalence; however, the most significant number of cases comes from areas with stable transmission such as Amazonia, the Pacific Coast, Antioquia, and Córdoba [4]. *Plasmodium vivax* and *P. falciparum* represent about 98% of all Colombian cases, and problems of plasmodial resistance to antimalarials or anophelines to insecticides do not explain their endemicity [5, 6].

The main goals of Colombia in the *National Malaria Strategic Plan 2019–2022* are reducing morbidity 40% by 2022 and mortality 80% by 2021. the *Plan of Action for the Elimination of Malaria 2016–202*0 of the Pan American Health Organization (PAHO), the aims are ***i)*** universal access to preventive interventions, vector control, diagnosis, and treatment; ***ii)*** strengthening of surveillance, health systems, planning, monitoring and evaluation of operational research; ***iii)*** facilitate elimination and prevent reintroduction into malaria-free areas [7].

On the other hand, malaria research in Colombia has been carried out mainly from a quantitative approach [8], framed in positivism and logical positivism. Positivism indicates that true knowledge is based on perceptible, sensorially verifiable facts, derived mainly from natural phenomena; while logical positivism or empiricism is a version applied to the philosophy of science, also called neopositivism, where scientific knowledge takes as the only criteria of validity the information empirically verifiable [9].

This research approach has been very useful for describing, explaining, and predicting different factors related to malaria, but it has limitations such as:

- explanation of the health-disease process in a fragmented way.
- predominance of linear approaches to identify risk factors.
- the exposure and vulnerability of subjects to different diseases is reduced to mathematical calculations (probabilistic) of different risks [10].

The above prevents grasping the conceptual and sociocultural complexity of the disease and accounting for other relevant realities for malaria control, which depend on the daily, cultural and social dynamics, and knowledge of the affected populations.

In this context, mixed studies, also called integrative, multi-method, or triangulation research, are a set of systematic, empirical, and critical research processes which involve the collection and analysis of quantitative and qualitative data, their integration and joint discussion in order to perform meta-inferences, and achieve a better understanding of the study phenomenon [11].

Mixed studies make it possible to overcome the limitations stated and enhance the strengths of quantitative (high sample sizes, evaluate trends, greater possibilities of generalization) and qualitative studies (refine details, delve into some categories) [12]. These studies articulate both approaches to broaden the knowledge of the problem [13], overcome dualistic views of reality for more dialectical ones [14], improve the scope of the results, and provide a more complete picture of the issue [15].

For public health, mixed methods are useful for the following reasons: ***i)*** they allow develop the public health as a transdiscipline that is not restricted by hegemonic theories and disciplinary knowledges and methods [16]; ***ii)*** grasp part of the complexity and diversity of the factors that influence health or determine the disease; ***iii)*** they enrich the results of traditional epidemiological through the generation of hypotheses, construction of sophisticated measures of social phenomena and explanation of results obtained through a quantitative approach [17]; ***iv)*** they are helpful in the assessment of practices, interventions, programs and policies in public health [18]; ***v)*** they are a common and frequent type of study in the field of community-based primary health care [19]; ***vi)*** improve the understanding of causal mechanisms and feedback of process, as well as the role of intersubjectivity in different interventions; ***vii)*** allow the simultaneity of a health practice-oriented to the subject and based on evidence [20]. Added to the above are various systematic reviews that have established these and other advantages in topics such as mental health [21], older adults [22], pregnant women with obesity [23], among others.

Specifically in malaria, there are studies with mixed methods that have evaluated strategies for case reporting [24], health knowledge and behaviors, attitudes towards vaccines, acceptability of different recommendations [25], patient satisfaction [26], among others. In Colombia, little is known about the use of mixed methods to study the disease, and systematic searches in Pubmed with the syntax *((mixed method [Title / Abstract]) AND (malaria [Title / Abstract])) AND (Colombia)*, in Scielo with *(ab: (mixed methods)) AND (ab: (malaria)) AND (Colombia)* and ScienceDirect with Colombia *Title, abstract, keywords: (mixed methods) AND malaria*, do not generate results.

For all the above, it is important to carry out a systematic review in this field in order to synthesize the available evidence in the country and determine its methodological quality; know the main topics addressed; identify ways to articulate quantitative and qualitative evidence on malaria; identify issues with consistent evidence to guide public policy actions; generate hypotheses on aspects not yet explored or explained in qualitative or quantitative research; broaden the understanding of the processes underlying the epidemiological profile of malaria in Colombia; and ultimately, to generate evidence that allows us to complement the actions of *National Strategic Plan for Malaria 2019–2022* from Colombia. In connection with these purposes, the objective of this research was to analyze the mixed studies on malaria in Colombia published in the world scientific literature between 1980–2022, through a broad approach of Cochrane, which one summarize the scientific production in an area, update a field of knowledge, summarize the available evidence, identify the main research topics or gaps in research, define future lines of work, among other aspects [27].

## Methods

### Type of study

A systematic review following PRISMA guideline [28]. The review was not registered and the protocol was not prepared.

### Study search and selection protocol

#### Identification

Through a pearl harvest [29] and a query in the thesauri DeCS (*in Spanish Descriptores en Ciencias de la salud*) and MeSH (*Medical Subject Headings*), the following search terms were identified: ***i)*** for the disease: malaria, *Plasmodium*, and Paludism; ***ii)*** for the method: mixed methods, qualitative, hermeneutic, ethnographies, ethnography, grounded theory, community-based participatory research, community-based research, participatory research, participatory action research, cultural anthropology, ethnopsychology. With these terms, nine search strategies were established and applied in the PubMed, OVID EMCare, Scielo, Science-Direct, Jstor, Web of Science, Campbell Collaboration / Cochrane Library, EMBASE, and HAPI databases (Table 1). Furthermore, three searches were carried out with the terms "interview", “focus group discussion” and "survey", which may have been more commonly used in some manuscripts that the general terms used to describe the qualitative or quantitative methodologies; without finding additional studies.

**Table 1:** Search syntax applied in databases

Source	Search strategy
**PubMed**	((Malaria[Title/Abstract] OR Plasmodium[Title/Abstract] OR Paludism[Title/Abstract]) AND mixed methods [Title/Abstract]) AND Colombia
	((Malaria[Title/Abstract] OR Plasmodium[Title/Abstract] OR Paludism[Title/Abstract]) AND qualitative[Title/Abstract]) AND Colombia
	((Malaria[Title/Abstract] OR Plasmodium[Title/Abstract] OR Paludism[Title/Abstract]) AND Hermeneutic[Title/Abstract]) AND Colombia
	((Malaria[Title/Abstract] OR Plasmodium[Title/Abstract] OR Paludism[Title/Abstract]) AND (Ethnographies[Title/Abstract] OR Ethnography[Title/Abstract])) AND (Colombia)
	((Malaria[Title/Abstract] OR Plasmodium[Title/Abstract] OR Paludism[Title/Abstract]) AND Grounded Theory[Title/Abstract]) AND Colombia
	((Malaria[Title/Abstract] OR Plasmodium[Title/Abstract] OR Paludism[Title/Abstract]) AND (Community-Based Participatory Research[Title/Abstract] OR community-based research[Title/Abstract])) AND Colombia
	((Malaria[Title/Abstract] OR Plasmodium[Title/Abstract] OR Paludism[Title/Abstract]) AND (Participatory Research[Title/Abstract] OR participatory action research[Title/Abstract])) AND Colombia
	((Malaria[Title/Abstract] OR Plasmodium[Title/Abstract] OR Paludism[Title/Abstract]) AND Cultural Anthropology[Title/Abstract]) AND Colombia
	((Malaria[Title/Abstract] OR Plasmodium[Title/Abstract] OR Paludism[Title/Abstract]) AND Ethnopsychology[Title/Abstract]) AND Colombia
**OVID EMCare**^**a**^	(((Malaria[Title/Abstract] OR Plasmodium[Title/Abstract] OR Paludism[Title/Abstract])) AND mixed methods[Title/Abstract]) AND Colombia
**Scielo**^**a**^	(ab:(Malaria OR Plasmodium OR Paludism)) AND (ab:( mixed methods)) AND (Colombia)
**Science-Direct**^**a**^	Colombia (Title, abstract, keywords: (malaria OR Plasmodium OR Paludism) AND mixed methods)
**Jstor**^**a**^	((ab:(Malaria OR Plasmodium OR Paludism) AND ab:( mixed methods)) AND (Colombia))
**Web of Science**^**a**^	TÍTULO: ((Malaria OR Plasmodium OR Paludism)) AND TÍTULO: (mixed methods) AND TEMA: (Colombia)
**Cochrane Library**^**a**^	Malaria OR Plasmodium OR Paludism in Title Abstract Keyword AND mixed methods in Title Abstract Keyword
**EMBASE**^**a**^	(malaria:ab,ti OR plasmodium:ab,ti OR paludism:ab,ti) AND mixed methods:ab,ti AND Colombia
**HAPI**^**a**^	Title: Malaria OR Plasmodium OR Paludism (and) Title: mixed methods (and) Subject: Colombia

^**a**^ The search with the term "mixed methods" is presented to show the specificity of the syntax of each source; In the remaining eight searches, the same strategy was applied, changing said term for the others concerning the method, as illustrated for PubMed

The searches were carried out in English and Spanish without time restrictions; the first time the search of literature was conducted on April 30, 2022 (the last update was carried out on December 1, 2022). They were complemented with searches for other publications in Google Scholar, Redalyc, and the system of libraries and institutional repositories of the main Colombian universities with research on malaria (Universidad de Antioquia, Universidad de Córdoba, Universidad del Valle, Universidad de Los Andes and Universidad de La Amazonía). It is important to note that the systematic review was limited to Colombia, since the findings of other studies in countries with a similar parasitological situation such as Venezuela, Peru or Brazil, are not comparable in key aspects of this review such as the following: type of system of health, type of disease control program, characteristics of the territory (physical, historical and cultural), political and social situation (with armed conflict, illegal mining, etc.), among other characteristics that may offer disparate results or that they are not applicable to the reality of Colombia. To manage references and eliminate duplicates we use Zotero.

#### Screening and eligibility criteria

We apply four inclusion criteria: include search terms in title, abstract, or keywords (the restriction to one or more of these three search fields, depended on the possible filters of each database consulted); be a study on malaria as the central outcome; developed in a Colombian population; and that it was original research. In this review we only applied two exclusion criteria: studies executed without a design based on mixed methods (investigations that only applied a quantitative or qualitative analysis), and studies with incomplete information or without data from the two components (Qual-Quant) of a mixed study on malaria. Two researchers conducted this phase independently and discrepancies were resolved by consensus.

#### Data extraction

Data extraction from the selected manuscript was carried out by extracting the following variables: title, authors, year of publication, study location, number and central characteristics of the study subjects, type of mixed study, data collection instruments, central theme or objective, central results of the quantitative component, categories of the qualitative component and conclusions. Then, qualitative synthesis was conducted. Two researchers also conducted this phase independently and discrepancies were resolved by consensus.

### Evaluation of methodological quality

Two researchers independently guaranteed the reproducibility of the studies' methodological quality assessment (disagreements were also resolved by consensus). The methodological rigor of the studies was evaluated based on the criteria of the guide "*Mixed Methods Appraisal Tool (MMAT)*" [30]. Given that the last item of the MMAT guide requires an independent assessment of each component, the criteria of the method section of the *STrengthening the Reporting of OBservational studies in Epidemiology (STROBE)* guidelines were applied for the quantitative component [31], because the results of the included studies showed that they were observational studies (despite not being explicit) and the *Standards for Reporting Qualitative Research (SRQR)* guidelines were applied for the qualitative component [32]. The supplementary data shows the assessments of the methodological quality of included studies, based on each of the items of the three evaluation guides (MMAT, STROBE and SRQR).

### Information analysis

Collected search results, screening, and data extraction process, were made in Excel. The percentage of studies that met each of the methodological quality criteria was determined, and for each study, the percentage of quality criteria met was determined. Because, in the quantitative component, the studies evaluated different units of analysis (dwellings, households, subjects, malaria registries) and studied heterogeneous subjects, it was not possible to carry out a quantitative synthesis (meta-analysis), but rather a qualitative one of the main variables, proportions, and temporal trends in malaria. In the qualitative component, the main categories reported from the studies were analyzed, and for each one, its properties (characteristics of a category that give it meaning) and dimensions (scale of variation of each property that gives it specificity and shows the variations of the study phenomenon) were identified.

Once the synthesis of the findings of each component was made, its articulation was carried out through a matrix in which the results were grouped in four levels:

1. The most investigated (proximal), called the epidemiological profile of malaria, includes aspects of morbidity, mortality, outbreaks, and knowledge surveys.
2. The second grouped the characteristics of the malaria control program after of the reform to the health system (Law 100 of 1993), giving an account of the impacts of this change on the operation and achievements of the program.
3. In the most profound or structural aspects, the changes in the Colombian health system in the 1990s appear.
4. The base of the hierarchy includes structural and permanent problems from Colombia in the last four decades, such as poverty; economic, social and political crises, and the neoliberal turn in the Colombian economic system.

Some approaches of the critical realism were taken to define these levels. Critical realism is an epistemological stance that allows overcoming the limitations of positivism and hermeneutics by making explicit the importance of investigating the simultaneity, interrelation, and superposition of the dimensions of reality (concrete totality): the empirical (observer's perceptions), historical, and transfactual (powers and structural determinants) [33].

## Result

### Selection and description of studies

**Fig. 1:**
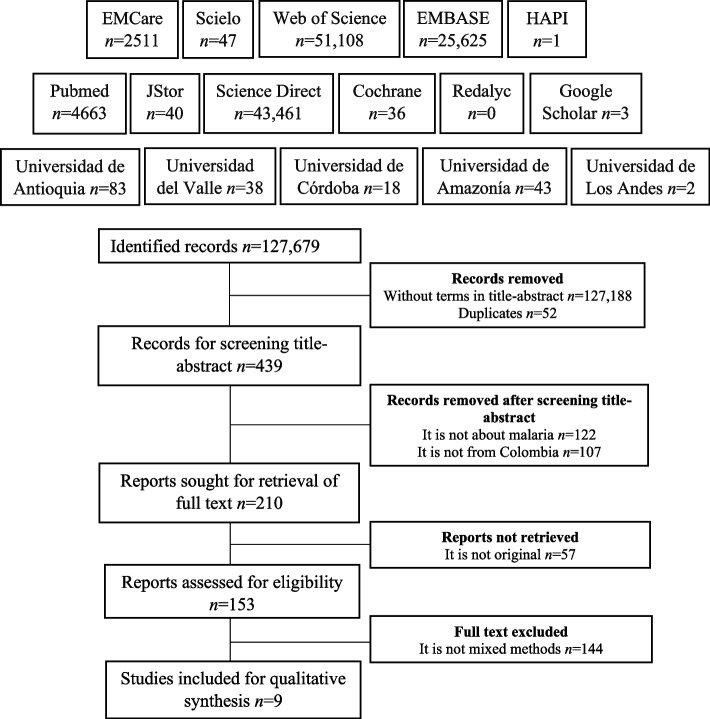
Search and selection of studies flow diagram

The search terms without restrictions at data bases (as recommended by PRISMA in the identification phase), allowed the identification of 127,679 records, of which 187 were identified in the manual search in Google Scholar (*n* = 91), Redalyc (*n* = 52), and the system of libraries and institutional repositories of the main Colombian universities (*n* = 44). Only 491 articles with search terms in title, abstract, or keywords were screened. Of the 153 eligible texts, 94 epidemiological studies (quantitative), ten entomological studies, 9 in vivo or in vitro models, 11 qualitative, and 20 corresponding to other typologies (modeling, economic evaluation, program analysis, etc.) were excluded. Therefore, only nine investigations that applied mixed methods were included (Fig. 1).

The studies were published between 1986 and 2018. Three were carried out in the department of Antioquia, one in Chocó, one in Amazonas, two on the Pacific Coast, and two were applied to the entire country. Only two investigations made explicit the mixed design used. The studies addressed the following topics: the importance of housing; knowledge, attitudes, and practices to design and implement a malaria control program; interaction of the disease with the nutritional and food situation; sustainability of the control policy in an endemic municipality; evaluation of the diagnostic network; and four investigations analyzed the impact of the reforms of the Colombian health system in the early 1990s on malaria control (Table 2).

**Table 2:** Description of the included studies according to year and study location, objective, and type of mixed design

Author	Year	Place	Objective	Design
Franco and TDR ^a^ [34]	1986	Necoclí	Describe the housing as an expression of economic, social, and cultural factors, and as a determinant of the malaria situation, 1982–1984	Unspecified (US)
Nieto et al. [35]	1999	Buenaventura	Study KAP ^**b**^ to design and implement a program with a PHC approach ^**c**^ for malaria control, 1993	US
Correa et al. [36]	2002	Basin of the Valle River, Chocó	Study the interaction of malaria, food and nutrition security, food and nutritional status, 2000–2001	US
Agudelo et al. [37]	2004	37 municipalities of 4 departments (Chocó, Nariño, Valle, Cauca)	Propose an alternative model (to the one established in Law 100 of 1993) for malaria control in municipalities and departments. Identify gaps in municipal management of the control program, 2002–2003	US
Cerón [38]	2004	Zaragoza, Tarazá, Apartadó, Turbo	Describe the malaria control model in the context of the GHSSS ^**d**^ (law 100–1993), 2003	US
Jiménez et al. [39]	2007	Throughout the country	Describe changes in malaria control related to reforms in the Colombian health policy 1982–2004	US
Knudson et al. [40]	2007	Throughout the country	Analyze the impact of the reforms carried out in the GHSSS ^**d**^ since the 1990s on the malaria situation and the actions to control the disease, 1970–2005	US
Salas et al. [41]	2014	El Bagre	Analyze the sustainability of the malaria control policy in the municipality of El Bagre, 2011	Dominant QUALI-quanti
Rondón and Tobón [42]	2018	Puerto Nariño and Tarapacá in the Amazonas	Characterize the operation of the epidemiological surveillance system (diagnostic network and its quality) for malaria in the Colombian-Peruvian border area, 2017	Conver-people parallel

^**a**^Special Program for Research and Training in Tropical Diseases

^**b**^KAP: Knowledge Attitudes and Practices

^**c**^PHC: Primary Health Care

^**d**^GHSSS: General Health Social Security System

In the qualitative component, all the studies used semi-structured interviews; one made explicit the use of observation [39] or field diary [36]; and three developed workshops or focus groups [36–38]. Several studies did not specify the number of participants, and among those that did, there were 117 interviews with people directly related to the malaria control program, including operational personnel, microscopists, health professionals; municipal, departmental, and national coordinators of the general vector control program and malaria program. In the quantitative component, 1729 subjects were included in whom were applied surveys about knowledge, attitudes, and practices on malaria [35, 36], perceived morbidity [36], all the studies inquired about institutional issues on the management of the disease, and six studies analyzed trends in morbidity or mortality from malaria [37–42]. Additionally, four studies [37, 39–41] supplemented their research with documentary reviews on regulations, programs, manuals, and previous researches on malaria control (Table 3).

**Table 3:** Description of the study populations in each component of the mixed design

Author	Typologies of investigation techniques used		
	**Qualitative**	**Quantitative**	**Documentary**
Franco and TDR, [34]	Interviews and field observations ^**a**^	Survey on housing conditions ^**a**^	None
Nieto et al. [35]	Five focus groups with community leaders ^**a**^	1380 surveys on KAP ^**b**^ of malaria	None
Correa et al. [36]	Interviews and workshops with four families ^**a**^	141 surveys of people dedicated to agricultural activities about KAP ^**b**^ of malaria, perceived morbidity and measurement of nutritional status	None
Agudelo et al. [37]	65 interviews with personnel from health secretariats and community and non-governmental organizations; 6 open interviews with members of the Malaria Eradication Service; 20 group meetings ^**a**^	Malaria morbidity and mortality trend since 1960; 102 surveys on institutional issues	19 municipals secretariats or Ministry documents 32 national or international documents on control programs
Cerón [38]	Interviews: departmental and municipal secretary of health, coordinator of departmental and municipal V-BD^**c**^, mayors Workshops in Villages with the community and auxiliary environmental health officials ^**a**^	Trends in malaria morbidity Surveys: departmental and municipal V-BD^**c**^ coordinator, municipal health care providing institutions, and insurance companies ^**a**^	None
Jiménez et al. [39]	Interview with three insurance officials	1982–2004 trend: cases (morbidity and mortality), annual parasite index, smears examined, and financial resources Survey of 55 community experts ^**d**^ and 26 institutional experts ^**e**^	Review of laws, regulations and manuals issued by the Ministry of Health, and by the Antioquia Sectional Health Directorate
Knudson et al. [40]	Interview with five experts in reforms and malaria, five managers of the V-BD^**c**^ program of the National Institute of Health, and five operational officials	Malaria statistics: incidence, financing, and resources	A systematic search of publications, studies, and investigations
Salas et al. [41]	Interviews with 14 people related to the malaria control program in the municipality ^**f**^	Measurement of malaria cases, gold price, armed confrontations, temperature, rainfall, and humidity in the municipality	Papers of journals, books and newspapers, and reports from governmental and non-governmental agencies
Rondón and Tobón [42]	Interviews with coordinators of malaria programs, public health surveillance (health personnel and community leaders), and the departmental laboratory of Public Health ^**a**^	They reported cases and questionnaires in the 25 diagnostic sites in Colombia	None

^**a**^Does not specify the number of subjects

^**b**^KAP: Knowledge Attitudes and Practices

^**c**^Vector-Borne Diseases

^**d**^Community leaders, homemakers, educators, farmers, miners, and students

^**e**^one from EPS, two from ESE (In Spanish: *Empresa Social del Estado*, or public hospital) managers, two from health care providing institutions managers, two from local health directors, four from officials and six from former officials of the departmental malaria program, one official from the Ministry of Social Protection, two international experts, three national experts, and three operational officials

^**f**^Officials of the Sectional Health Secretariat of Antioquia and El Bagre, the Pan American Health Organization, bacteriologists, microscopists, and people hired through the Fundación Universidad de Antioquia

## Methodological quality

**Fig. 2:**
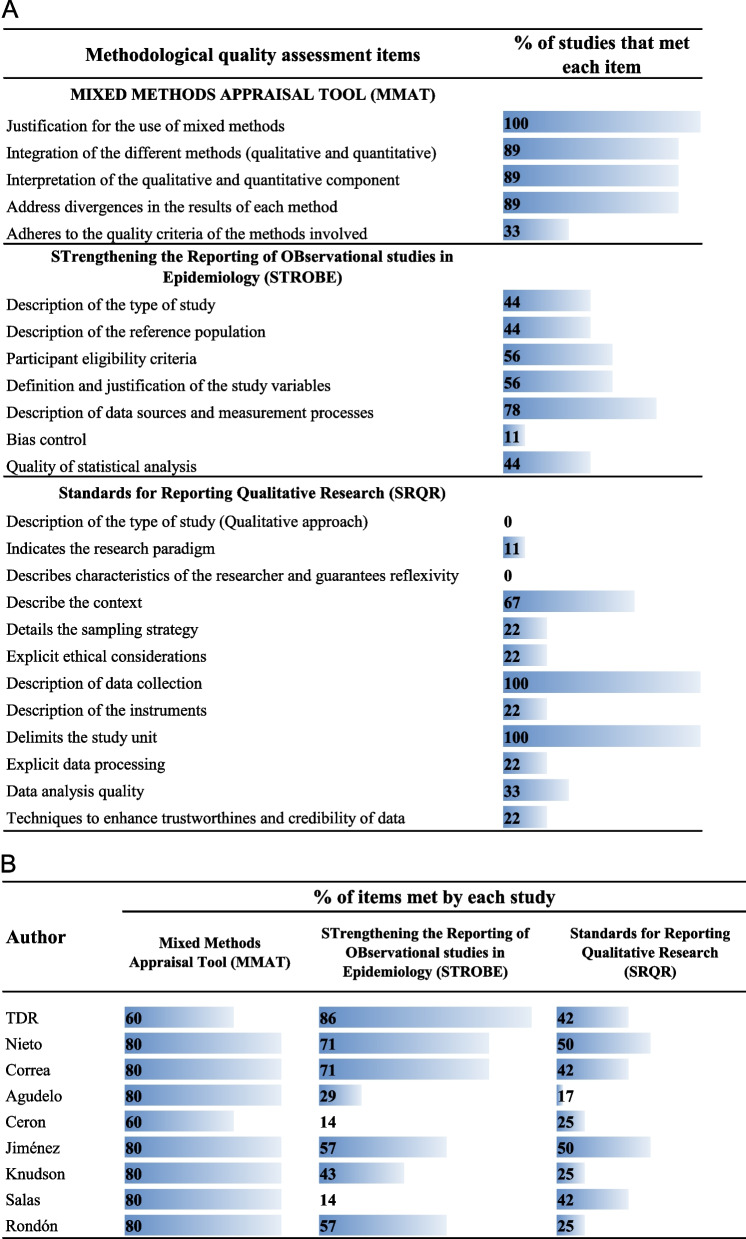
Assessment of the methodological quality of the included studies. **A** Evaluation of each quality item. **B** Evaluation of each study

In the quality evaluation 33% of the studies applied at least half of the quality criteria of each component (quantitative or qualitative). In this sense, most studies did not correctly describe the quality criteria applied in their quantitative component, being bias control the least applied. In the qualitative component, the least applied items were the specification of the type of study and the guarantee of reflexivity (Fig. 2).

### Central findings quantitative component

Information related to units of analysis such as housing and families in endemic areas, exposed subjects, and control programs were obtained:

- *Housing:* important characteristics are reported, such as poor material conditions of the floor, ceiling, and walls, as well as the absence of sanitary infrastructure in most of the cases analyzed (greater than 70%) [34].
- *Families:* in this unit stands out the high proportion of poverty (according to the material conditions of life, but without giving a precise definition of this term), the high risk of greater impoverishment due to the disease, and due to the type of economic activity of its members (primarily people who are paid for each workday), absence of protection mechanisms against malaria (mosquito nets, insecticides or others) and overcrowding [34]. The extended family with minors, agricultural vocation, and self-subsistence predominates (production, distribution, and consumption at the interior of the village); approximately one third (30%) with food insecurity, and 69% of children with global and chronic malnutrition and 31% with acute malnutrition [36].
- *Subjects exposed to malaria*: the largest proportion does not attend hospitals because they are far from their homes, the long waiting times, the poor-quality care (friendliness), and delay in diagnosis [35]. There are a high proportion (> 70%) of subjects with history of previous malaria- Also, vector and main symptoms of malaria were frequently identified [36].
- *Malaria control program at the municipal, departmental or national level*: the following findings are highlighted: ***i)*** the national trends evaluated since 1960 show that mortality has decreased and morbidity has increased, with an underestimation of cases between 15–25%, and with the presence of outbreaks in some years [37]; ***ii)*** in the decade 1991–2000, the highest incidence occurred in the Department of Antioquia (one of those that reports the highest proportion of cases in the country), the highest peaks occurred between 1992 and 1995 with 17 municipalities concentrating 75% of all the Department's cases [34, 35]; ***iii)*** Antioquia, like the national average, shows a decrease in mortality from 1946 to 2003 [38, 39], although other authors show that it increased rapidly after 1986 [37]. After 1993, morbidity continued to increase and began to express itself in a set of related problems: repeated outbreaks, urban malaria, and other related problems [37].

However, morbidity trends should be analyzed with caution due to the following problems in data availability: decrease and lack of knowledge over several years of the number of blood samples examined [39]; underreporting of cases that affect the numerators (couple with variation in data from some sources of the surveillance system); abrupt and unjustified changes in the official reports on the populations exposed or at risk, for example, in 2002 it was 22.4 million, in 2003 it was 8.3 million, and in 2004 it was 11.4 million [40]. Nevertheless, those responsible indicate that figures prior to 2002 were inflated because they considered that only 10–15 municipalities in the country had urban malaria [40], although this research does not delve into these aspects.

Added to the above is a recent study showing a low diagnostic and epidemiological surveillance capacity in some areas. For example, the Rondón study with 25 diagnostic sites in southern Colombia indicates that less than 30% had microscopy stations; approximately half of them performed the diagnosis for malaria, and the rest only took the sample and sent it to a reference laboratory; the Secretariat of Health of the Amazon (Colombia) does not carry out monitoring visits, and only 60% of the diagnostic points participate in quality assurance programs [42]. However, it should be noted that this region is not part of the endemic areas with the highest number of cases and exposed population, but it does reflect the problems of surveillance of the event in areas far from urban areas.

### General findings of the qualitative component

In this component, issues related to the concept of health were found in some exposed subjects from rural areas, socioeconomic characterization of some studied regions, the role of the State, and impacts of the change in Colombia's general health social security system in 1993.

Regarding the conceptions of health of some endemic areas' residents, it was mentioned that health is related to biological, psychological, social, and environmental factors. At the same time, the disease is explained in terms of socioeconomic problems reflected in the absence of sewerage, little supply of water and food, among others. Also, nosological systems that group diseases into infectious (diarrhea, malaria, skin infections, and intestinal parasites being the most important), behaviors that affect health (such as violence and poor personal hygiene), pests (rodents, mosquitoes, cockroaches), and environmental problems (absence of sewerage, garbage disposal, drinking water, or adequate shelter) were found [35, 36].

In describing the socioeconomic context, some authors found that migration to urban areas to seek better education and income generates problems of food insecurity [36]. This is connected with a study that compared socioeconomic context in the rural and urban areas of an endemic municipality in the Department of Antioquia. The urban area is characterized by more significant commercial activity, public services, transportation facilities, and diversity in the economic activities of members of the household; while rural area is characterized by a rural economy, high emigration, low land tenure, a workforce employed mainly in agricultural farming, and the exchange of products with the village neighbors [34].

In some testimonies, the importance of having a strong State was also highlighted, even though the reality of the residents of some endemic areas of the Department of Antioquia, mainly in the rural area, shows the low presence of the State, the failure to comply with its responsibilities in security, its inability to control armed groups, low coverage of basic health-care services, problems (and in some cases absence) of road infrastructure, few incentives for agricultural production and low inclusion of social organizations in activities related to managing their health [34].

Finally, various categories were found that account for the negative effects of changes in the Colombian general health social security system at the end of the 1980s and the beginning of the 1990s, from the perspective of key actors such as experts from the Pan American Organization of Health, the National Institute of Health, the departmental and local health secretariats, as well as operational personnel and community leaders. In this sense, it is indicated that since 1993 there have been radical changes in the structure and organization of public health programs and in the functions or responsibilities of territorial entities, which resulted in fewer investments in core public health issues, and loss of the capacity and experience gained in previous decades. Also, significant territorial gaps in planning, allocation of resources, personnel, training, disease monitoring, and intersectoral coordination are outlined [37].

Although the most significant impacts have been recorded since 1993, several authors highlight that the significant changes related to the competencies of territorial entities, particularly the decentralization (of the departments in 1994 and the municipalities in 1997) that affected public health programs, had been a constant and long-term process in Colombia since 1986, modeled by Laws 12 of 1986, 10 of 1990, 60 of 1993, 715 of 2001, being a milestone Law 100 of 1993 that completely reformed the health system [37, 38]. In addition to these normative resources, other conditions that affected the different public health programs are added, such as economic and political crises, changes from care models towards insurance models, the transition from State to private, the delegation of the public health service, and the autonomy of health institutions [40].

### Qualitative findings regarding malaria and its control

Malaria was classified as one of the most critical health problems among the subjects studied. The main symptoms of malaria, the causative agent, the vector, and the prevention methods (use mosquito nets, avoid puddles, and not plant trees near the house) are identified. The subjects know that the vector lives and reproduces in stagnant waters, that transmission occurs by mosquito bites, that the tropical climate partly explains the disease's presence, and that the mosquito is found in bushes, near garbage, stagnant water swamps and latrines. Knowledge is often not directly related to practice; many people start self-medicating since no prescription is required in pharmacies [35, 36].

People living in endemic areas go to health services (malaria post, health center, or hospital) or traditional healers. They make the initial (symptomatic) diagnosis at home and manage it with medicinal plants, although as the fever progresses, they visit the health center for pharmacological treatment [36]. Most subjects expressed more confidence in health institutions and pharmacological treatment than traditional medicine; the latter is used only at the onset of symptoms [35]. Some authors allude to deficiencies in physical infrastructure and medical equipment, making it difficult to give an adequate diagnosis and timely notification of cases. Despite this, it is essential to highlight that microscopists have a good reputation, and among the decision-makers and the community, they are trusted for their performance [41].

In addition to cultural practices and beliefs, the primary socio-ecological dynamics related to malaria include mining, migration, armed conflict, and climatic variations [41]. In addition, increases in morbidity, mainly due to outbreaks, are attributed to climate change, social conditions (armed conflict, migration), deterioration of preventive actions, and financing problems of the control program [40]. The studies also highlight activities for social participation to identify and intervene risk factors through community health committees, malaria management committees, and intersectoral activities at the municipal level with education, public services, mining companies, and other key actors [38].

Finally, in this component it is highlighted the testimonies of key actors and the documentary research on the negative impacts that the changes in the General Health Social Security System had on the malaria control program in Colombia, highlighting the following events:

i. Loss of progress made since the Malariology Campaign from 1943 and the Malaria Eradication Service (in Spanish SEM) from 1956, in terms of community education and training, active case detection [37], operational and administrative efficiency in the network of diagnosis and treatment, integrated vector control and epidemiological surveillance of the event [38, 39].
ii. An economic emphasis of the program (focused on billing, costs, and cost–benefit relationships), with a prevalence of privatization in disease care and an individualistic view of health [40].
iii. Disarticulation and fragmentation of responsibilities and preventive actions, low integration with local health plans, and little participation of the community and local authorities in control activities [40].
iv. Since 1991, a deterioration in malaria control has been observed, associated with decentralization and the subjection of health policy to market principles, with progressive weakening of State responsibilities, dismantling of installed capacity, loss of know-how and experience acquired, fragmentation of control actions, and the collapse of the information system [39].
v. Reduction of economic resources allocated to the program, subordination of health care to insurance contracts, delayed payments for health service providers, and little clarity on the allocation and administration of resources [37, 39, 40].
vi. Organizational problems since the health secretariats are subject to political influence, with little participation of the personnel and an unstable relationship with institutions of training. Additionally, there is personnel without sufficient technical skills, low salary, corruption in hiring, little horizontal and vertical articulation with other vector control programs, and delays in providing drugs, mosquito nets, and insecticides [37].

### Articulation of quantitative and qualitative findings

**Fig. 3:**
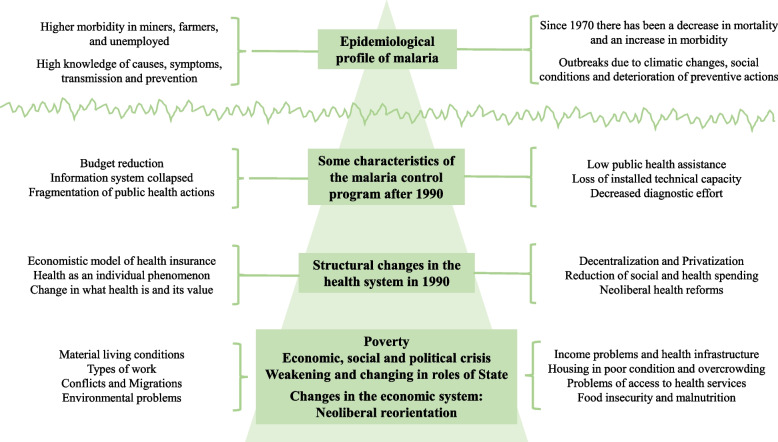
Articulation and hierarchical organization of the main findings from the systematized mixed studies

The previous results account for proximal, intermediate, and structural processes associated with malaria in Colombia. Using some postulates of critical realism (Fig. 3), the evidence from the mixed studies allows to account for four levels:

i. The first would correspond to the most visible part or to the proximal processes, closest to the clinical-epidemiological outcome, which materialize in the *clinical-epidemiological profile* of malaria. This level includes the findings of the quantitative component of the studies that show increasing trends in morbidity, the presence of outbreaks, groups with a higher prevalence of the event, and high levels of knowledge about the causes, symptoms, transmission, treatment and prevention of malaria.
ii. The second level shows the *characteristics of the malaria control program* after the changes introduced with the health reforms that began in the late 1980s, highlighting the reduction in the program's budget, problems with information systems and surveillance, fragmentation of malaria control actions, among others.
iii. The previous characteristics are based on the changes derived from implementing the new social security health law (Law 100 of 1993), whose axes were the economic vision of health, decentralization, privatization, the reduction of social spending, among other neoliberal policies.
iv. Finally, all the previous characteristics underlie problems related to the neoliberal economic system, the change in the role of the State, poverty, and the social, economic and political crises that the country has experienced (Fig. 3).

Figure 3 shows that the phenomenon's dimensions that are generally observed correspond to a small fraction; most of what happens with the problem studied is below the surface. In dialectical terms, it would be stated as follows: the superficial is shaped by the final effects (empirical research), but its explanatory factors and determination processes are deep, complex, and dialectical, requiring other types of knowledge (theoretical, of common thought and the everyday world) to explain and understand the phenomenon of study [33, 43].

## Discussion

Through 9 search strategies applied in 15 sources, the study found nine mixed studies on malaria in Colombia published between 1986 and 2018 in various topics and units of analysis. This low number of studies accounts for several important aspects:

- It confirms that in malaria research in Colombia, the biomedical studies focused on the parasite, the vector and clinical-epidemiological aspects predominate [8];
- It coincides with previous systematic reviews that demonstrate the low academic-scientific production in qualitative studies and social processes that underlie malaria [44, 45];
- This predominance of approaches of traditional epidemiology will not allow progress in the intervention of the malaria in Colombia since its conception of health is limited in theoretical and practical terms [46].

Regarding the last aspect, it is worth noting that, in health philosophy and social epidemiology, this phenomenon has been called the "paradigm in crisis" of classical epidemiology. This phenomenon occurs because their reasoning and methods do not address all levels of reality (it is limited to the individual or singular level with little development of the particular and general levels); they assume the population only as the sum of individuals (with a short and limited approach of the causal mechanisms of population problems), favoring the measurement of biological factors, behavioral factors or external environmental exposures; they do not allow addressing social, historical and cultural aspects of the health-disease process; they generate partial public policy recommendations given the limitations of their ontological, epistemological and methodological foundations; and they have low explanatory power for key outcomes such as morbidity or mortality [46].

The above highlights the importance of articulating the quantitative tradition with qualitative findings that make it possible to overcome some of the limitations mentioned above. In this sense, this systematic review shows the advantages of mixed studies by revealing social, economic, political, cultural, and historical causes, generally not revealed in the hegemonic research on malaria in Colombia. Thus, the advantages of mixed methods materialize, such as capturing part of the complexity and diversity of the factors that determine health disease; enriching the results of traditional epidemiological studies [17]; improving the evaluation of public health practices, interventions, programs, and policies [18]; articulate-involve community knowledge and intersubjectivity in health actions [19, 20]; among others. Despite these advantages, it is essential to improve the description of methods in these designs, since, in this review, the methodological quality was regular, mainly because criteria that guarantee the validity and rigor of this type of research were not made explicit (although it is appropriate to indicate that the standardization of the rigor criteria of the guidelines used is posterior to most of the studies included in this review).

In the quantitative and qualitative findings, it was common the reference to homes, families, and socioeconomic context characterized by poverty and its related problems such as migration (mainly of inhabitants of rural areas seeking better opportunities or a floating population such as miners), food insecurity, barriers to access to health services and the increased risk of becoming ill. This complexity of the social and economic contexts in which the disease occurs has been widely documented for general issues that relate to poverty, malnutrition, and infectious diseases [47]. These also constitute findings that have been documented for many years for the specific case of malaria, thus demonstrating the bidirectionality and feedback between malaria and poverty, with multiple and complex causal mechanisms that need to be studied in the specificity of each affected population [48]. Despite this multiplicity of connection mechanisms between both problems (for example, the bad health conditions of the migrant population, the presence of greater barriers to access to health services among the poor, delay in diagnosis, greater risk of suffering severe malaria, etc.), it is clear that effective malaria control requires intervening malaria per se and simultaneously reducing poverty, which is to say that as long as poverty is not intervened, it will be impossible to control malaria and many other infectious diseases [48, 49].

In this regard, it is essential to clarify that the studies in this review that refer to poverty are not exhaustive or precise in the use of this concept. In this sense, it is important to note that in the study of poverty, there are multiple visions (needs, standard of living, insufficient resources, violation in the exercise of rights), approaches (absolute poverty without reference to the socioeconomic context, or relative poverty), definitions (from economics, sociology, philosophy), methods (focused on preferences, material wealth, capabilities), and measurement typologies (univariate or multivariate) demonstrating that this field of study is complex. In the case of malaria in Colombia, it is a subject that has not been rigorously investigated, which should be corrected in subsequent studies [50].

On the other hand, it is essential to note that the social and practical representations that subjects and communities have regarding malaria are part of a broader sociocultural system referred to social constructions on health and its value, and they determine the initial actions against health, disease care, hospital consultation and the type of treatment accessed. The finding from the systematized studies in this research coincides with a previous review on the qualitative studies of malaria in Colombia, in which it is established that, from the perspective of social actors, their conception of the health system revolves around the following categories: ***i)*** social representations, meanings, perceptions, wisdom, experiences, and practices about the health-disease process; ***ii)*** nosology, knowledge, beliefs and therapeutic itineraries of the traditional health system; and ***iii)*** the meanings of the health-disease process as a material, cultural and spiritual reality [45]. These aspects are fundamental in designing and evaluating disease control initiatives with social participation and recognition of the community heritage.

In this regard, it was also found that the knowledge, attitudes, and practices about the causality, transmission, treatment, and prevention of malaria are satisfactory. This finding indirectly reflects the effect of the education, communication, and health information campaigns that different institutions and community groups have carried out on the disease as part of the objectives of control programs since 1957, which has resulted in the incorporation of a part of biomedical knowledge in the communities [37, 38]. The above differs from that reported in other systematic reviews on this topic in Asia; In them, it was reported that the general community and some health professionals lacked general knowledge and awareness about the disease, its transmission, and control and prevention measures [51].

In the structural aspects that determine the profile of malaria, multiple testimonies captured in the qualitative component highlight two interrelated factors, the low presence of the State (security, health care, road, and sanitary infrastructure, social inclusion) and the armed conflict derived from this, findings that coincide with that reported in a systematic review of qualitative studies carried out in Colombia [45]. The nexus between health disease and welfare state policies (understood as a set of government actions to seek greater attention to the redistribution and general welfare of the population) has been widely documented. For example, it has been reported that these types of states, mainly those with egalitarian ideologies, tend to implement redistributive policies; implementing policies aimed at reducing social inequalities, which have resulted in a reduction in infant mortality and an increase in life expectancy at birth; and made it possible to improve public health profiles and their interaction with employment policies during the twentieth century [52–54].

The results of this research also show that the establishment of neoliberal policies in Colombia, at the end of the 1980s and during the 1990s, negatively affected public health in general and malaria control in particular, because it implied the reduction of the budget, fragmentation of the information system, disarticulation of control actions, reduction of active surveillance of cases, reduction of the diagnostic effort, among other effects derived from the change from a healthcare model to an insurance model, adjusted to the logic of private markets and decentralization. These findings coincide with previous publications that have documented multiple characteristics of neoliberalism in health, highlighting the positioning of the private sector for health care or "privatization of individual health"; the lack of financing of the public sector and the dissemination of the idea of its inefficiency to justify the participation of private health companies; assume the provision of health services as an axis of capitalist accumulation; promote the use of generally expensive technologies that result in the need to accept private capital; outsource hospital processes; give technocratic management to the health system; prioritize economic efficiency mainly by reducing costs and medical personnel; and not addressing issues of health inequities as a priority [55–57].

### Limitations

Despite the fact that some narratives of the qualitative component relate the changes in the health system with the economic model (mainly with neoliberalism), the evidence on this relationship is not solid or consistent, which requires further investigation. In general, the structural or deep determinants of the epidemiological profile of malaria in Colombia were only captured in the qualitative component, which has limitations in its inference; For this reason, more quantitative and mixed studies are required to analyze these findings in greater detail.

## Conclusion

This research shows that health education and communication efforts have effectively generated a significant amount of knowledge about the etiology, symptoms, treatment, and prevention of the disease. It also shows that malaria morbidity has not been effectively impacted in the last decades. The epidemiological profile of malaria morbidity, from traditional epidemiology, has been sustained by environmental problems, armed conflict, individual risk behaviors, and low adherence to recommendations from health institutions. However, the qualitative component of the mixed studies reveals deeper causes that are less studied, of greater theoretical complexity, and that reflect greater challenges to design and implement health interventions, such as socioeconomic and political crises, poverty, and the neoliberal orientation in the policy of malaria control; The latter reflected in the change in the role of the State, the fragmentation of control actions, the predominance of insurance over social assistance, the privatization of the provision of health services, an individualistic and economistic predominance of health, low connection with the popular heritage and the social and community initiatives, among others. These characteristics show the importance of expanding mixed studies as a source of evidence to improve malaria research and control models in Colombia and identify the underlying causes of the epidemiological profile. The intervention of the underlying causes is not being included in current control policies, plans, and projects.

## Supplementary Information

**Additional file 1.**

## Data Availability

All data generated or analyzed during this study are included in this published article [and its supplementary information files].
